# Draft Genome Sequence of the Fluconazole-Resistant Yarrowia lipolytica Clinical Isolate CBS 18115

**DOI:** 10.1128/mra.01260-22

**Published:** 2023-03-02

**Authors:** Rose-Anne Lavergne, Paul Barbier, Lenha Mobuchon, Atanu Banerjee, Rajendra Prasad, Florent Morio

**Affiliations:** a Nantes Université, CHU Nantes, Cibles et Médicaments des Infections et de l'Immunité, UR1155, Nantes, France; b Service de Virologie, CHU Nantes, Nantes, France; c Nantes Université, CHU Nantes, Biology Department, Nantes, France; d Amity Institute of Biotechnology, Amity University Haryana, Gurugram, India; e Amity Institute of Integrative Sciences and Health, Amity University Haryana, Gurugram, India; University of California, Riverside

## Abstract

Yarrowia lipolytica is a nonconventional yeast of industrial interest that can sometimes act as an opportunistic pathogen and is responsible for invasive fungal infections. We report the draft genome sequence of the fluconazole-resistant strain CBS 18115, which was isolated from a blood culture. The Y132F substitution in *ERG11*, which was previously described in fluconazole-resistant *Candida* isolates, was identified.

## ANNOUNCEMENT

Yarrowia lipolytica belongs to the *Ascomycota* phylum, *Saccharomycotina* subphylum, and *Dipodascaceae* family (1). In addition to its industrial use (2), Y. lipolytica is widespread in food, the environment, and animals (1). Due to an inconstant ability to grow above 32°C, this species is generally regarded as safe for industrial use (1). Yarrowia lipolytica is an opportunistic pathogen that can causes invasive candidiasis (3). *In vitro*, this species is considered susceptible to fluconazole (4). The first Y. lipolytica genome (CLIB122) was published in 2004 (5). We report the draft genome sequence of a fluconazole-resistant Y. lipolytica clinical isolate that was collected from a blood culture following surgery for ulcerative colitis. Interestingly, despite previous azole exposure, this strain displayed a fluconazole MIC of >256 μg/mL using a gradient concentration strip method (Etest; bioMérieux). The patient was successfully treated with caspofungin.

This strain was grown on a chromogenic agar plate (CAN2; bioMérieux) at 35°C, and identification was obtained with a Vitek matrix-assisted laser desorption ionization–time of flight mass spectrometry (MALDI-TOF MS) instrument (bioMérieux). Following lyticase cell wall digestion, genomic DNA was extracted using the QIAmp DNA minikit (Qiagen). Libraries were constructed using the Illumina DNA preparation tagmentation kit (Illumina). Briefly, 30 ng of total DNA was fragmented and indexed using the bead-linked transposome technology and the Integrated DNA Technologies (IDT) for Illumina DNA/RNA unique dual (UD) indexes set. Libraries were amplified, purified, and quantified using the Qubit high-sensitivity kit (Thermo Fisher Scientific). Finally, 9 pM pooled and denatured libraries were put in a 2 × 250-bp v2 kit (Illumina) and sequenced with a MiSeq instrument (Illumina).

The raw reads were trimmed, assembled into contigs, and scaffolded using Trim Reads v2.5 and *De Novo* Assembly v1.5 tools from the CLC Genomics Workbench v22.0 (Qiagen). Contigs with coverage of <10× and lengths of <500 bp were discarded using coverage-versus-length plots (6). The final set of scaffolds was quality analyzed using QUAST v5.0.2 (7).

The total genome size was 20,255,408 bp, distributed over 521 scaffolds (coverage of 100×), with an *N*_50_ value of 105 kbp (longest scaffold, 397 kbp) and a GC content of 49.03%. AUGUSTUS v3.4.0 (8) predicted 6,151 protein-coding genes using a Candida albicans training data set, and 484 tRNA genes were detected using tRNAscan-SE 2.0 (9). Genome completeness was estimated as 95.3% using BUSCO v5.3.2 and the saccharomycetes_odb10 lineage data set (10). An average nucleotide identity (ANI) calculation performed with the ANI Comparison tool (CLC Genomics Workbench; Qiagen) indicated 99.72% similarity to the genome of CLIB122 (GenBank accession number GCF_000002525) (5).

The strain’s putative *ERG11* gene (encoding the target of azole drugs), which was identified using the protein BLAST algorithm, yielded a protein of 523 amino acids that showed 59.21% similarity to its homolog in C. albicans (GenBank accession number X13296). Interestingly, our isolate differed from CLIB122 (GenBank accession number XP_500518.1) by a single mismatch at residue 132 (Y132F). Because this substitution has been associated with acquired fluconazole resistance in distant yeast species, we can expect its contribution to the elevated fluconazole MIC of our strain (11, 12). Given the contributions of ATP-binding cassette (ABC) transporters to multidrug resistance in fungal species (13), we also identified the putative ABC proteins in our isolate. Our analysis with a previously reported pipeline (14) revealed 32 ABC proteins, of which 26 were predicted to be transporters, based on the presence of transmembrane helices. Interestingly, at least 5 probable pleiotropic drug resistance subfamily transporters were identified.

### Data availability.

This isolate has been deposited at the Westerdijk Fungal Biodiversity Institute under identification number CBS 18115. This whole-genome shotgun project has been deposited in DDBJ/ENA/GenBank under the accession number JAPPVN000000000, and the raw reads have been deposited under SRA accession number SRX17434142. These data are also available under BioProject accession number PRJNA877082. All parameters used for sequence assembly and analysis are available at https://doi.org/10.6084/m9.figshare.21342630.v1.
